# Diagnostic Principles for Chronic Gastritis Associated with Duodenogastric Reflux

**DOI:** 10.3390/diagnostics13020186

**Published:** 2023-01-04

**Authors:** Maria A. Livzan, Sergei I. Mozgovoi, Olga V. Gaus, Dmitry S. Bordin, Alexei V. Kononov

**Affiliations:** 1 Department of Faculty Therapy and Gastroenterology, Omsk State Medical University, 644099 Omsk, Russia; 2 Department of Pathological Anatomy, Omsk State Medical University, 644099 Omsk, Russia; 3 A.S. Loginov Moscow Clinical Scientific Center, Department of Pathology of the Pancreas, Biliary Tract and Upper Digestive Tract, 111123 Moscow, Russia; 4 Department of Propaedeutics of Internal Diseases and Gastroenterology, A.I. Yevdokimov Moscow State University of Medicine and Dentistry, 127473 Moscow, Russia; 5 Department of General Medical Practice and Family Medicine, Tver State Medical University, 170100 Tver, Russia

**Keywords:** chronic gastritis, biliary gastritis, duodenogastric reflux, reactive gastropathy, bile acids

## Abstract

This article systematizes available data from the literature on biliary gastritis (BG) in order to increase the awareness of specialists about the latest possibilities for diagnosing the disease. BG occurs as a result of pathological duodenogastric reflux. In patients with a preserved duodenogastric junction, the dominant factor is represented by motor disorders of the upper digestive tract (primary biliary gastritis), while in patients recovering from surgical interventions it is represented by structural changes (secondary biliary gastritis). Progressive BG can lead to atrophy of the gastric mucosa, intestinal metaplasia, epithelial dysplasia, and eventually to gastric cancer. Diagnostic methods for BG are carried out to identify risk factors, exclude alarm symptoms and identify persistent motor disorders and pathological reflux (24 h pH-impedancemetry, hepatobiliary scintigraphy, 24 h monitoring of bilirubin content in the reflux using a Bilitec 2000 photometer), as well as to diagnose gastritis itself (esophagogastroduodenoscopy, morphological gastrobiopsy examination). The diagnosis of BG should be based on a multidisciplinary approach that combines a thorough analysis of a patient’s complaints, an anamnesis of the disease, and the results of endoscopic and histological research methods.

## 1. Introduction

Chronic gastritis is now understood as a group of chronic diseases that are morphologically characterized by persistent inflammatory infiltrate and impaired cell renewal with the development of intestinal metaplasia, atrophy, and epithelial dysplasia in the gastric mucosa [1]. According to the data of recent epidemiological studies, there has been a decrease in the proportion of *Helicobacter pylori*-associated gastritis with an increase in the contribution of other etiological factors. Thus, the prevalence of gastritis associated with bile reflux ranges from 17% to 24%, reaching 62% in a cohort of patients with risk factors for duodenogastric reflux [2,3].

Historically, the study of biliary gastritis (BG) as well as other types of gastritis became possible only at the beginning of the 20th century after the introduction of endoscopic examination into routine clinical practice. However, one of the first mentions in medical literature of a burning sensation in the epigastrium and heartburn possibly associated with bile reflux belongs to the Scottish doctor William Buchan and dates back to 1784. In his work “Domestic medicine (or a treatise on the prevention and cure of disease by regimen and simple medicines)”, he wrote “What is commonly called the heartburn, is not a disease of that organ, but an uneasy sensation of heart, or acrimony, about the pit of the stomach, which is sometimes attended with anxiety, nausea, and vomiting. It may proceed from debility of the stomach, indigestion, bile, the abounding of an acid in the stomach…” [4].

Biliary or duodenogastric reflux (from Latin *refluo*—“flow back”; hereafter—DGR) refers to the retrograde reflux of duodenal contents, including bile and pancreatic enzymes, into the stomach. Biliary reflux can also be observed under physiological conditions, in the early morning hours, in the postprandial period and against the background of prolonged fasting. With an increase in the frequency of refluxes and/or the duration of contact of the aggressive components of the refluxate with the gastric mucosa (GM), damage to the latter and the development of BG may occur (synonyms include “reflux gastritis”, “reactive gastropathy”, “chemical gastritis”, “gastropathy associated with bile reflux”) [5]. Thus, BG occurs as a result of pathological duodenogastric reflux.

The assessment of the diagnostic possibilities and development mechanisms of BG is extremely relevant not only due to the possible risk of developing atrophy or intestinal metaplasia, but also because of the frequent association of this type of gastritis with a more severe course of gastroesophageal reflux disease. When gastroesophageal reflux occurs, the refluxate components include bile and pancreatic juice too [6].

This article systematizes the available data from the literature on BG in order to increase the awareness of specialists about the latest possibilities for diagnosing the disease. At first, the main available databases (Pubmed/Medline, Embase and Google Scholar) were examined in search of relevant literature. Key terms such as “biliary gastritis”, “gastropathy associated with bile reflux”, “duodenogastric reflux” were used to search for articles. The articles were selected according to the following criteria: full-text articles published in English through October 2022, including original studies, systematic reviews and meta-analyses. No geographic restrictions were applied.

## 2. Risk Factors for The Formation of BG

### 2.1. Factors Associated with Age, Gender and Type of Constitution

The incidence of BG is reported to be higher among young and old people when compared with middle-aged people. In addition, BG is less common in men than in women [7]. In general, the highest incidence is observed among young women. From a clinical point of view, it is important to note that BG patients are often tall and lean. It is assumed that an important role in this is played by gastroptosis, which is often found among people with an asthenic type of constitution and associated gastric motility disorders [8].

### 2.2. Factors Associated with Lifestyle

Smoking not only has a direct damaging effect on the gastric mucosa but also causes relaxation of the pyloric sphincter, which contributes to the reverse flow of bile into the stomach [9]. Alcohol is an independent etiological factor associated with the development of reactive gastropathy [1], and in the case of duodenogastric reflux it can potentiate the damaging effect of fatty acids on the gastric mucosa [9].

Eating habits such as excessive consumption of sugary foods and coarse dietary fiber are also associated with the risk of developing BG. Sweet carbonated drinks stimulate the secretion of glucagon and cholecystokinin, hormones that suppress gastric motility [10]. Coarse dietary fiber helps to relax the fundus of the stomach and slows down gastric emptying, which reduces the efficiency of bile clearance from the stomach.

### 2.3. Factors Associated with Anatomical Changes in the Area of the Duodenogastric Junction

The duodenogastric junction is a physiological barrier that prevents retrograde reflux of bile into the stomach. Anatomical changes that contribute to pathological DGR are usually associated with such previous surgical interventions as gastric resection, gastroenterostomy, sleeve gastrectomy, enterostomy, pyloroplasty, etc. [11,12].

### 2.4. Factors Associated with Persistent Motor Disorders

These factors Include Pathology of the Biliary Tract, Cholelithiasis, including in the Period after Cholecystectomy, Parasitic and Helminthic Invasions [13]. In Addition, Motor Disorders of the Upper Digestive Tract May Be Associated with Metabolic Diseases (Diabetes Mellitus, Obesity) [14,15].

In patients with a preserved duodenogastric junction, the dominant factor is represented by motor disorders of the upper digestive tract (primary biliary gastritis), and in patients after surgical interventions it is represented by structural changes (secondary biliary gastritis).

Despite the fact that BG is a separate type of gastritis, in real clinical practice it is possible to combine it with other types of gastritis. According to separate epidemiological studies, the frequency of detection of the main etiological factor in the development of gastritis—*Helicobacter pylori* (*H. pylori*) infection—in BG patients is lower than in the general population [16]. In contrast, others have reported that the prevalence of *H. pylori* in patients with BG is similar to or greater than that in healthy individuals [17]. On the one hand, *H. pylori* infection can increase the secretion of gastrin, which reduces the peristalsis of the antrum of the stomach and contributes to the development of DGR. On the other hand, it is believed that a change in the pH of the stomach to the alkaline side against the background of DGR leads to a violation of the mucosal–bicarbonate barrier and creates unfavorable conditions for the survival and colonization of *H. pylori*. In addition, bile acids in high concentrations have their own bactericidal action against *H. pylori* [16].

## 3. Mechanisms for the Development of BG

The composition of the duodenal contents that enter the lumen of the stomach with DGR includes bile acids and pancreatic juice (amylase, lipase, elastase, nuclease, carboxylase, trypsinogen, chymotrypsinogen). Bile acids (BAs) are the main components of bile and, according to their chemical structure, are derivatives of cholanic acid. Primary fatty acids—cholic and chenodeoxycholic—are synthesized from cholesterol in hepatocytes. In the composition of bile, primary fatty acids are in the form of conjugates—compounds with the amino acids glycine or taurine [18]. Postprandial contraction of the gallbladder promotes the release of bile into the duodenum, where, with the active participation of bile acids, fats are emulsified, pancreatic lipase is activated, and a number of hydrophobic substances (cholesterol, fat-soluble vitamins, vegetable steroids, etc.) are absorbed. Having reached the terminal ileum, some of the conjugated primary BAs are released from glycine and taurine and enter the liver with the portal blood flow; some reach the large intestine and, under the action of 7-α-hydroxylase of bacteria, are dehydroxylated with the formation of secondary BAs, lithocholic and deoxycholic [19,20]. Deoxycholic acid is absorbed by passive diffusion and continues to participate in the enterohepatic circulation along with primary fatty acids, while lithocholic acid, owing to its poor solubility, is not reabsorbed and is excreted in the feces [21]. The total pool of fatty acids in humans in one cycle of enterohepatic circulation is 2–4 g, and the cycle itself is repeated 7–10 times a day, as a result of which up to 30 g of fatty acids are absorbed per day, while about 10% is lost with feces [22].

Fatty acids and their salts are known to exert the main damaging effect on the coolant. This happens through a number of mechanisms:

- Lysolecithin, which is formed from lecithin under the action of phospholipase A, destroys the phospholipid layer of the cell membranes of the coolant epithelium;
- Inhibition of the nitric oxide synthetase enzyme activity results in DNA damage, apoptosis, and cell mutation;
- Increased back diffusion of H+, stimulation of mast cells and, as a consequence, a greater release of histamine occur. As a result, there is an increase in the secretion of hydrochloric acid, which is not only a factor of aggression in relation to the coolant, but also potentiates the negative effect of the bile acids themselves [23,24].

It is also important that, at a low pH, BAs change, acquiring even more cytotoxic properties, which cause penetration through cell membranes, damaging intercellular contacts [25].

An increase in gastrin secretion by G cells under conditions of DGR is associated not only with an increase in hydrochloric acid production but also with the inhibition of pyloric sphincter contractions [26].

The pathogenesis of BG can also be considered through the prism of an imbalance between aggression and defense factors. The predominance of aggression factors leads to a decrease in the resistance of the mucous membrane of the stomach with the development of a syndrome of increased epithelial permeability, which contributes to the penetration of bacterial antigens and toxins into the submucosal layer and the development of an inflammatory response [27]. In turn, the degradation products of eosinophils and mast cells of the inflammatory infiltrate leads to the irritation of afferent neurons, to visceral hypersensitivity and, as a result, to the development of pain and motor disorders [28].

One of the most pressing issues today is the study of the qualitative and quantitative composition of the microbiota of the digestive tract, in particular the stomach. The microbiota of the digestive tract is a highly dynamic structure that can change over time in response to various changes. Recently, publications have appeared, demonstrating the modulation of the gastric microbiota against the background of a change in gastric pH caused by DGR [29]. A study by Wang S. et al. demonstrated that the excess content of BAs in the contents of the stomach is associated with an increased number of bacteria that produce lipopolysaccharides, for example, *Prevotella melaninogenica* (*P. melaninogenica*) [30]. *P. melaninogenica* is known to induce inflammation by activating the IL-6/JAK1/STAT3 pathway, leading to additional damage to the gastric mucosa. It is also important that the amount of *P. melaninogenica* is significantly increased in tumor tissue samples from gastric cancer, which confirms the role of the microbiota in carcinogenesis [31]. A study with a very small sample (10 people with intestinal metaplasia and 10 healthy people) examined differences in the microbiome of the stomach and duodenum. The study concluded that the diversity of the duodenal microbiota in the control group was higher than in that with intestinal metaplasia. Moreover, there was a significant difference in the qualitative composition of the microbiota between the groups. In particular, *Lactococcus*, *Flavobacterium*, *Psychrobacter*, *Mysroides*, *Enhydrobacter*, *Streptococcus* and *Leuconostoc* were found in higher numbers in patients with intestinal metaplasia. At the same time, the presence or absence of *H. pylori* did not affect the composition of the duodenal microbiota. Based on the results of this study, it follows that in cases of DGR, bacteria enter the stomach under physiological conditions and live in the lumen of the duodenum. Colonization of the mucosal surface with bacteria that are not characteristic of the gastric microbiota induces inflammation in it, which enhances the pathogenic effect of the bile acids themselves (synergistic effect) [32]. Progressive BG can lead to atrophy of the gastric mucosa, intestinal metaplasia, epithelial dysplasia and eventually to gastric cancer [33,34]. In their study, Matsuhisa T. et al. demonstrated that the development of intestinal metaplasia due to exposure to high concentrations of fatty acids does not depend on the presence of *H. pylori* [35]. In addition, under the influence of fatty acids and their salts, even after successful eradication therapy, the function of the mucosal barrier remains impaired with a change in the microRNA profile [36]. The combination of pathological DGR and *H. pylori* infection has also been reported to increase the risk of development and rate of progression of gastric cancer [37]. In addition to direct cellular cytotoxicity, increasing evidence points to the interaction of BAs with their nuclear and membrane receptors as additional factors influencing the risk of gastric cancer [38].

**Figure 1:**
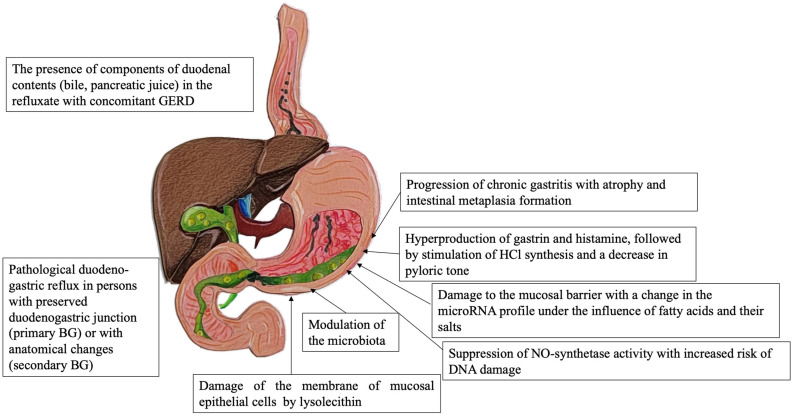
The main correlations in the pathogenesis of BG. BG—biliary gastritis, GERD—gastroesophageal reflux disease, BA—bile acids, GM—gastric mucosa.

The regurgitation of aggressive duodenal contents into the gastric cavity may disrupt the gastric mucosa barrier and damage the mucosal cells. Long-term reflux results in accelerated regeneration of the gastric epithelium. High concentrations of bile acids were shown to have an effect on the induction and progression of intestinal metaplasia. This process can be implemented by the production of caudal-related homeobox family 2 (CDX2) and mucin 2 (MUC2) expression via FXR/NF-κB signaling [39] and cyclooxygenase-2 (COX-2) expression [40]. As a result, these molecular–biological events lead to gastric intestinal metaplasia. Acidic bile salts can induce telomerase activity via the c-Myc-dependent mechanism [41]. Nevertheless, several studies have demonstrated the anticarcinogenic effects of bile acids in gastric cancer [42,43]. The main correlations in the pathogenesis of BG are shown in Figure 1.

## 4. Stages of Diagnosing BG

The clinical picture of BG consists of dyspepsia syndrome, as well as symptoms associated with the pathology of the biliary tract. Some patients may be asymptomatic [44].

The patient may complain of “hungry pains” in the epigastrium or in the pyloroduodenal zone, which are often accompanied by a feeling of bitterness in the mouth or belching of bitter contents. This is due to the influence of the aggressive components of bile in the composition of the refluxate. Postprandial distress syndrome, manifested by a feeling of heaviness in the epigastrium after eating and rapid satiety, nausea, belching of air and decreased appetite, may develop against a background of severe motor-evacuation disorders.

It should be noted that there are reports of an association between the severity of dyspepsia syndrome and the concentration of bile components (bilirubin) in samples of gastric juice. For example, a study by Keighley M.R.B. et al. showed that patients with a bilirubin content in samples exceeding 1 mg/100 mL had more pronounced symptoms, and severe endoscopic changes were detected more often [45].

There is also evidence that the course of gastroesophageal reflux disease (GERD) in patients with BG may be associated with more pronounced clinical symptoms, frequent erosions in the esophageal mucosa and a complicated course of the disease [46,47]. In addition, it has been shown that about 88% of patients with GERD are refractory to standard therapy with proton pump inhibitors when mixed reflux is present [48].

For diagnostic purposes it is important to identify the risk factors for BG (female gender, young or old age, asthenic physique, eating habits with excessive consumption of sweets, saturated fats, coarse fiber, obesity, concomitant pathology of the hepatobiliary system, diabetes mellitus, stomach and/or gallbladder surgery, etc.) and to collect the anamnesis of the disease from the emergence of the first symptoms, as well as to clarify the chronological relationship between the identified risk factors and the onset of BG. To exclude the impact of other etiological factors of gastritis, primarily alcohol and drug-induced gastritis, it is necessary to carefully collect an anamnesis regarding alcohol consumption and use of drugs that can damage the gastric mucosa, non-steroidal anti-inflammatory drugs, antiplatelet agents, anticoagulants, glucocorticosteroids, etc.

Methods for a non-invasive diagnosis of BG include complete blood count, biochemical blood test, coprological examination, the analysis of markers of helminthic and parasitic invasions and ultrasound examination of the abdominal organs; such tests are carried out to identify risk factors as well as to exclude alarm symptoms and concomitant diseases.

Invasive methods of the instrumental diagnosis of BG are aimed at identifying persistent motor disorders and pathological reflux (24 h pH-impedancemetry, hepatobiliary scintigraphy, 24 h monitoring of bilirubin content in the reflux using a Bilitec 2000 photometer), as well as diagnosing gastritis itself (esophagogastroduodenoscopy, morphological gastrobiopsy examination). None of these methods for diagnosing pathological reflux should be considered a recommended guideline, as each of them has both its advantages and disadvantages [49].

- The main method for diagnosing pathological DGR at present is 24 h pH-impedancemetry. Daily pH-impedancemetry allows for differential diagnosis between physiological and pathological GDR, quantitative analysis of episodes of acidic, weakly acidic, and non-acidic reflux [50]. The method is invasive and has high sensitivity but low specificity.
- Hepatobiliary scintigraphy is a method for diagnosing DGR based on the determination of the content of the stomach after a choleretic breakfast of a radiopharmaceutical excreted in the bile, administered intravenously [51]. The test is well-tolerated by patients, while the method has high sensitivity and specificity; however, the high cost and radiation effects on the body limit its use in clinical practice. Among the shortcomings of this method, it should be noted that the volume and composition of the refluxate cannot be determined.
- The bilirubin content in the refluxate is monitored for 24 h using a Bilitec 2000 photometer. In vitro studies have demonstrated a statistically significant relationship between the concentration of bilirubin and bile acids in the refluxate, which allows us to consider bilirubin an alternative marker of DGR [52]. Using bilirubin as a DGR marker, the Bilitec 2000 allows recording the frequency of these refluxes and the duration of bile residence in the stomach; however, the measurement result can be affected by changes in pH and dilution of the refluxate.

For the diagnosis of any type of gastritis, conducting an esophagogastroduodenoscopy (EGDS) is mandatory, then followed by the morphological study of the gastrobiopsy specimens. EGDS has high sensitivity and specificity for the diagnosis of gastritis and is accompanied in the primary diagnosis by a targeted biopsy of the gastric mucosa in five points and the assessment of each of them according to the Operative Link for Gastritis Assessment system. This contributes to a more accurate prediction of the course of gastritis and refers to the methods of cancer prevention [53].

There are no pathognomonic macro- or microscopic signs of BG. The endoscopic picture and histological changes actually coincide with those of a gastropathy associated with the use of non-steroidal anti-inflammatory drugs. It is important to remember that visualization of bile reflux into the stomach during endoscopic examination as well as the presence of non-specific morphological signs indicating damage to the epithelial barrier are not sufficient to diagnose a gastritis caused by bile reflux.

**Figure 2:**
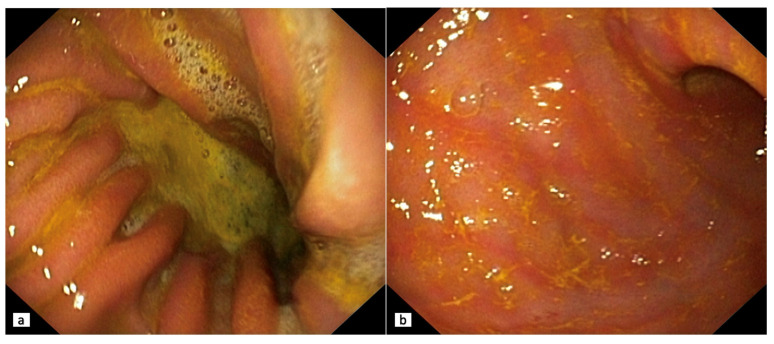
(**a**) Duodenogastric reflux. Endoscopic observation of gastric mucosa (white light endoscopy) in patient A, 53 years old, with a history of cholecystectomy (2 years before the study). The gastric folds are thickened, the mucosa is loose, swollen, and unevenly hyperemic, mainly on the peaks of the folds. Refluxate is a heterogeneous viscous bile that is associated with mucosa and is poorly washed. (**b**) Endoscopic observation (white light endoscopy) in patient T., 58 years old. Bile reflux gastritis. The lower third of the stomach is represented by linear hyperemia, swelling of the folds. Stomach contents are viscous, heterogeneous bile, which is removable only when forced water supply. After washing the bile mucosa is swollen with petechial hemorrhages. The patient has a history of non-alcoholic fatty liver disease and cholelithiasis.

As a rule, endoscopic manifestations in BG are represented by hyperemia and edema of the mucous membrane which spread circularly from the pyloric region in the proximal direction (Figure 2). At the same time, bile spots are often found on the surface of the coolant, and there is a visible reflux of bile from the duodenum into the stomach or a high content of bile in the lumen of the stomach [54].

**Figure 3:**
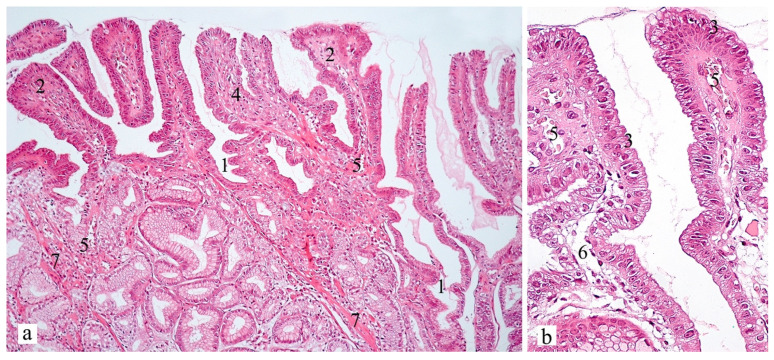
Gastric antral mucosa in patient A, 59 years old. Hematoxylin and eosin, (**a**) ×100, (**b**) ×400. Signs of foveolar hyperplasia with the presence of tortuous gastric pits (corkscrew appearance, (1)) and papillary deformity of the surface (2). Decreased mucus-producing activity of mucocytes with hyperchromic enlarged nuclei (reactive nuclei, (3)). In the lamina propria there is a scanty mononuclear infiltrate (4), the presence of ectatic blood vessels (5), edema (6). An increase in the number (*hyperplasia*) of *cells* of muscularis mucosa with the formation of branching fibers surrounding the glands (7) can be noted.

Microscopic signs of BG include weak diffuse, predominantly mononuclear inflammatory infiltration of the lamina propria and pronounced regenerative changes in the epithelium (foveolar hyperplasia with hyperchromic cell nuclei, decreased mucus formation, presence of corkscrew-shaped tortuous gastric pits). Furthermore, the mucosa is characterized by edema with ectatic blood vessels in the lamina propria and the presence of subnuclear vacuolization of the cytoplasm, which is a morphological phenomenon associated with the operated stomach syndrome. In the lamina propria, there are bundles of smooth muscle cells that divide the glands into groups (Figure 3).

## 5. Conclusions

BG can develop as a result of changes in the biliary tract and pyloroduodenal zone after surgical interventions, as well as in individuals with functional disorders of the upper digestive tract motility without a history of previous operations. In turn, the failure of the sphincter apparatus, antroduodenal discoordination and deterioration of the properties of the bile itself cause the formation of a recurrent reflux of the contents of the duodenum into the stomach, with damage to its mucous membrane. It is known that BAs with detergent properties directly damage the GM and also potentiate the negative impact of other aggressive components (*H. pylori*, gastric juice) on it.

There are no specific clinical, endoscopic or histological features in BG. Difficulties in the diagnostic process are due to the need to identify, on the one hand, structural changes in the gastric mucosa qualified as “gastritis” and, on the other hand, pathological duodeno-gastric reflux. In this case, a differential diagnosis is carried out among other etiological factors of the group of chemical gastritis (gastropathy). The lack of widespread introduction of highly valid tests for the detection of pathological biliary reflux into routine practice should perhaps be considered the most significant limitation.

The diagnosis of BG is obtained based on a comprehensive assessment of the objective status and anamnesis data, primarily with regard to concomitant diseases of the biliary tract as well as previous surgical interventions on the abdominal organs that may contribute to the formation of DGR. In the absence of pronounced signs of chronic inflammation, the presence of reactive changes in the mucosa and the colonization of *H. pylori* against the background of a characteristic endoscopic picture (hyperemia and edema of the mucous membrane of the pyloroduodenal zone, bile spots on the surface) can be considered evidence of BG.

## Data Availability

Not applicable.

## References

[1] Sugano K., Tack J., Kuipers E.J., Graham D.Y., El-Omar E.M., Miura S., Haruma K., Asaka M., Uemura N., Malfertheiner P. (2015). Kyoto global consensus report on Helicobacter pylori gastritis. Gut.

[2] Taşcı E.K., Karakoyun M., Sezak M., Doğanavsargil B., Çetin F., Aydoğdu S. (2022). Does bile reflux reduce Helicobacter pylori gastritis?. Turk. J. Pediatr..

[3] Basnayake C., Geeraerts A., Pauwels A., Koek G., Vaezi M., Vanuytsel T., Tack J. (2021). Systematic review: Duodenogastroesophageal (biliary) reflux prevalence, symptoms, oesophageal lesions and treatment. Aliment. Pharmacol. Ther..

[4] Buchan W., Strahan W., Cadell T., Balfour J., Creech W. (1784). Domestic Medicine (Or a Treatise on the Prevention and Cure of Disease by Regimen and Simple Medicines).

[5] Keane F.B., Dimagno E.P., Malagelada J.R. (1981). Duodenogastric reflux in humans: Its relationship to fasting antroduodenal motility and gastric, pancreatic, and biliary secretion. Gastroenterology.

[6] Tack J. (2005). Review article: Role of pepsin and bile in gastro-oesophageal reflux disease. Aliment. Pharmacol. Ther..

[7] Li D., Zhang J., Yao W.Z., Zhang D.L., Feng C.C., He Q., Lv H.H., Cao Y.P., Wang J., Qi Y. (2020). The relationship between gastric cancer, its precancerous lesions and bile reflux: A retrospective study. J. Dig. Dis..

[8] Shi X., Chen Z., Yang Y., Yan S. (2022). Bile Reflux Gastritis: Insights into Pathogenesis, Relevant Factors, Carcinomatous Risk, Diagnosis, and Management. Gastroenterol. Res. Pract..

[9] Maity P., Biswas K., Roy S., Banerjee R.K., Bandyopadhyay U. (2003). Smoking and the pathogenesis of gastroduodenal ulcer--recent mechanistic update. Mol. Cell. Biochem..

[10] Bihter Gürler E., Özbeyli D., Buzcu H., Bayraktar S., Carus İ., Dağ B., Geriş Y., Jeral S., Yeğen B.Ç. (2017). Natural sweetener agave inhibits gastric emptying in rats by a cholecystokinin-2- and glucagon like peptide-1 receptor-dependent mechanism. Food Funct..

[11] Aprea G., Canfora A., Ferronetti A., Giugliano A., Guida F., Braun A., Battaglini Ciciriello M., Tovecci F., Mastrobuoni G., Cardin F. (2012). Morpho-functional gastric pre-and post-operative changes in elderly patients undergoing laparoscopic cholecystectomy for gallstone related disease. BMC Surg..

[12] Zobolas B., Sakorafas G.H., Kouroukli I., Glynatsis M., Peros G., Bramis J. (2006). Alkaline reflux gastritis: Early and late results of surgery. World J. Surg..

[13] McCabe M.E., Dilly C.K. (2018). New Causes for the Old Problem of Bile Reflux Gastritis. Clin. Gastroenterol. Hepatol..

[14] Watkins C.C., Sawa A., Jaffrey S., Blackshaw S., Barrow R.K., Snyder S.H., Ferris C.D. (2000). Insulin restores neuronal nitric oxide synthase expression and function that is lost in diabetic gastropathy. J. Clin. Investig..

[15] Yamamoto S., Watabe K., Takehara T. (2012). Is obesity a new risk factor for gastritis?. Digestion.

[16] Kawai Y., Tazuma S., Inoue M. (2001). Bile acid reflux and possible inhibition of Helicobacter pylori infection in subjects without gastric surgery. Dig. Dis. Sci..

[17] Manifold D.K., Anggiansah A., Rowe I., Sanderson J.D., Chinyama C.N., Owen W.J. (2001). Gastro-oesophageal reflux and duodenogastric reflux before and after eradication in Helicobacter pylori gastritis. Eur. J. Gastroenterol. Hepatol..

[18] Liu H., Hu C., Zhang X., Jia W. (2018). Role of gut microbiota, bile acids and their cross-talk in the effects of bariatric surgery on obesity and type 2 diabetes. J. Diabetes Investig..

[19] Stellaard F., Sackmann M., Sauerbruch T., Paumgartner G. (1984). Simultaneous determination of cholic acid and chenodeoxycholic acid pool sizes and fractional turnover rates in human serum using 13C-labeled bile acids. J. Lipid Res..

[20] Režen T., Rozman D., Kovács T., Kovács P., Sipos A., Bai P., Mikó E. (2022). The role of bile acids in carcinogenesis. Cell. Mol. Life Sci..

[21] Zwartjes MS Z., Gerdes VE A., Nieuwdorp M. (2021). The Role of Gut Microbiota and Its Produced Metabolites in Obesity, Dyslipidemia, Adipocyte Dysfunction, and Its Interventions. Metabolites.

[22] Dawson P.A. (2011). Role of the intestinal bile acid transporters in bile acid and drug disposition. Handb. Exp. Pharmacol..

[23] Goldman A.S., Hahidullah M., Goldman D., Khailova L., Watts G., Delamere N., Dvorak K. (2010). A novel mechanism of acid and bile acid-induced DNA damage involving Na^+^/H^+^ exchanger: Implication for Barrett’s oesophagus. Gut.

[24] Bechi P., Amorosi A., Mazzanti R., Dei R., Bianchi S., Mugnai L., Masini E. (1993). Reflux-related gastric mucosal injury is associated with increased mucosal histamine content in humans. Gastroenterology.

[25] Choi J., Kim S.G., Yoon H., Im J.P., Kim J.S., Kim W.H., Jung H.C. (2014). Eradication of Helicobacter pylori after endoscopic resection of gastric tumors does not reduce incidence of metachronous gastric carcinoma. Clin. Gastroenterol. Hepatol..

[26] He Q., Liu L., Wei J., Jiang J., Rong Z., Chen X., Zhao J., Jiang K. (2022). Roles and action mechanisms of bile acid-induced gastric intestinal metaplasia: A review. Cell Death Discov..

[27] Oshima T., Miwa H. (2016). Gastrointestinal mucosal barrier function and diseases. J. Gastroenterol..

[28] Camilleri M. (2019). Leaky gut: Mechanisms, measurement and clinical implications in humans. Gut.

[29] Igarashi M., Nakae H., Matsuoka T., Takahashi S., Hisada T., Tomita J., Koga Y. (2017). Alteration in the gastric microbiota and its restoration by probiotics in patients with functional dyspepsia. BMJ Open Gastroenterol..

[30] Wang C., Li W., Wang H., Ma Y., Zhao X., Zhang X., Yang H., Qian J., Li J. (2019). Saccharomyces boulardii alleviates ulcerative colitis carcinogenesis in mice by reducing TNF-α and IL-6 levels and functions and by rebalancing intestinal microbiota. BMC Microbiol..

[31] Liu X., Shao L., Liu X., Ji F., Mei Y., Cheng Y., Liu F., Yan C., Li L., Ling Z. (2019). Alterations of gastric mucosal microbiota across different stomach microhabitats in a cohort of 276 patients with gastric cancer. EBioMedicine.

[32] Gong J., Li L., Zuo X., Li Y. (2019). Change of the duodenal mucosa-associated microbiota is related to intestinal metaplasia. BMC Microbiol..

[33] Straub D., Oude Elferink R.P., Jansen P.L., Bergman J.J., Parikh K., Krishnadath K.K. (2019). Glyco-conjugated bile acids drive the initial metaplastic gland formation from multi-layered glands through crypt-fission in a murine model. PLoS ONE.

[34] Matsuhisa T., Tsukui T. (2012). Relation between reflux of bile acids into the stomach and gastric mucosal atrophy, intestinal metaplasia in biopsy specimens. J. Clin. Biochem. Nutr..

[35] Matsuhisa T., Arakawa T., Watanabe T., Tokutomi T., Sakurai K., Okamura S., Chono S., Kamada T., Sugiyama A., Fujimura Y. (2013). Relation between bile acid reflux into the stomach and the risk of atrophic gastritis and intestinal metaplasia: A multicenter study of 2283 cases. Dig. Endosc..

[36] Takahashi Y., Uno K., Iijima K., Abe Y., Koike T., Asano N., Asanuma K., Shimosegawa T. (2018). Acidic bile salts induces mucosal barrier dysfunction through *let-7a* reduction during gastric carcinogenesis after *Helicobacter pylori* eradication. Oncotarget.

[37] Li X.B., Lu H., Chen H.M., Chen X.Y., Ge Z.Z. (2008). Role of bile reflux and Helicobacter pylori infection on inflammation of gastric remnant after distal gastrectomy. J. Dig. Dis..

[38] Rohr M., Aljabban J., Rudeski-Rohr T., Lessans S., Nakkina S.P., Hadley D., Zhu X., Altomare D.A. (2021). Meta-Analysis Reveals the Prognostic Relevance of Nuclear and Membrane-Associated Bile Acid Receptors in Gastric Cancer. Clin. Transl. Gastroenterol..

[39] Yu J.H., Zheng J.B., Qi J., Yang K., Wu Y.H., Wang K., Wang C.B., Sun X.J. (2019). Bile acids promote gastric intestinal metaplasia by upregulating CDX2 and MUC2 expression via the FXR/NF-κB signalling pathway. Int. J. Oncol..

[40] Park M.J., Kim K.H., Kim H.Y., Kim K., Cheong J. (2008). Bile acid induces expression of COX-2 through the homeodomain transcription factor CDX1 and orphan nuclear receptor SHP in human gastric cancer cells. Carcinogenesis.

[41] Ni Z., Min Y., Han C., Yuan T., Lu W., Ashktorab H., Smoot D.T., Wu Q., Wu J., Zeng W. (2020). TGR5-HNF4alpha axis contributes to bile acid-induced gastric intestinal metaplasia markers expression. Cell Death Discov..

[42] Yang H.B., Song W., Cheng M.D., Fan H.F., Gu X., Qiao Y., Lu X., Yu R.H., Chen L.Y. (2015). Deoxycholic acid inhibits the growth of BGC-823 gastric carcinoma cells via a p53-mediated pathway. Mol. Med. Rep..

[43] Song W., Yang H.B., Chen P., Wang S.M., Zhao L.P., Xu W.H., Fan H.F., Gu X., Chen L.Y. (2013). Apoptosis of human gastric carcinoma SGC-7901 induced by deoxycholic acid via the mitochondrial dependent pathway. Appl. Biochem. Biotechnol..

[44] Lake A., Rao SS C., Larion S., Spartz H., Kavuri S. (2021). Bile Reflux Gastropathy and Functional Dyspepsia. J. Neurogastroenterol. Motil..

[45] Keighley M.R., Asquith P., Alexander-Williams J. (1975). Duodenogastric reflux: A cause of gastric mucosal hyperaemia and symptoms after operations for peptic ulceration. Gut.

[46] Vaezi M.F., Richter J.E. (1999). Importance of duodeno-gastro-esophageal reflux in the medical outpatient practice. Hepato-Gastroenterol..

[47] Naik R.D., Meyers M.H., Vaezi M.F. (2020). Treatment of Refractory Gastroesophageal Reflux Disease. Gastroenterol. Hepatol..

[48] Kunsch S., Neesse A., Linhart T., Nell C., Gress T.M., Ellenrieder V. (2012). Prospective evaluation of duodenogastroesophageal reflux in gastroesophageal reflux disease patients refractory to proton pump inhibitor therapy. Digestion.

[49] Eldredge T.A., Myers J.C., Kiroff G.K., Shenfine J. (2018). Detecting Bile Reflux-the Enigma of Bariatric Surgery. Obes. Surg..

[50] Barrett M.W., Myers J.C., Watson D.I., Jamieson G.G. (1999). Dietary interference with the use of Bilitec to assess bile reflux. Dis. Esophagus.

[51] Saarinen T., Pietiläinen K.H., Loimaala A., Ihalainen T., Sammalkorpi H., Penttilä A., Juuti A. (2020). Bile Reflux is a Common Finding in the Gastric Pouch After One Anastomosis Gastric Bypass. Obes. Surg..

[52] Shenouda M.M., Harb S.E., Mikhail SA A., Mokhtar S.M., Osman AM A., Wassef AT S., Rizkallah NN H., Milad N.M., Anis S.E., Nabil T.M. (2018). Bile Gastritis Following Laparoscopic Single Anastomosis Gastric Bypass: Pilot Study to Assess Significance of Bilirubin Level in Gastric Aspirate. Obes. Surg..

[53] Pimentel-Nunes P., Libânio D., Marcos-Pinto R., Areia M., Leja M., Esposito G., Garrido M., Kikuste I., Megraud F., Matysiak-Budnik T. (2019). Management of epithelial precancerous conditions and lesions in the stomach (MAPS II): European Society of Gastrointestinal Endoscopy (ESGE), European Helicobacter and Microbiota Study Group (EHMSG), European Society of Pathology (ESP), and Sociedade Portuguesa de Endoscopia Digestiva (SPED) guideline update 2019. Endoscopy.

[54] Chang W.K., Lin C.K., Chuan D.C., Chao Y.C. (2016). Duodenogastric Reflux: Proposed New Endoscopic Classification in Symptomatic Patients. J. Med. Sci..

